# Endobronchial Carcinoid and Concurrent Carcinoid Syndrome in an Adolescent Female

**DOI:** 10.1155/2016/2074970

**Published:** 2016-11-08

**Authors:** Jonathan D. Cogen, Jonathan Swanson, Thida Ong

**Affiliations:** ^1^Division of Pulmonary Medicine, Department of Pediatrics, University of Washington, Seattle, WA, USA; ^2^Division of Pediatric Radiology, Department of Radiology, University of Washington, Seattle, WA, USA

## Abstract

Endobronchial carcinoid tumors are the most common intrabronchial tumors in children and adolescents. Common signs and symptoms include persistent cough and wheezing not responsive to bronchodilators, hemoptysis, and recurrent fever. Diagnosis is frequently made by imaging and direct visualization with flexible bronchoscopy; surgery remains the gold standard treatment, and lung-sparing resections should be performed whenever possible. Though carcinoid syndrome—characterized by flushing, palpitations, wheezing, shortness of breath, and diarrhea—has been found in association with adult bronchial carcinoid tumors, to our knowledge only one previous study has reported the presence of carcinoid syndrome in a pediatric patient with an endobronchial carcinoid. Here, we report a case of a 14-year-old girl with chronic cough found to have an endobronchial carcinoid tumor and signs and symptoms consistent with carcinoid syndrome.

## 1. Introduction

Pulmonary tumors in the pediatric population are rare and account for only 0.2% of all cases of childhood cancer [1]. Carcinoid tumors are malignant neuroendocrine tumors and, together with pleuropulmonary blastomas, make up the most common pediatric pulmonary malignancies [2]. Carcinoid syndrome—a result of serotonin overproduction from a functional neuroendocrine tumor, leading to systemic complaints of flushing, bronchospasm, diarrhea, and edema [3]—has been reported in association with adult bronchial carcinoids, though, to our knowledge, no previous reports have described the presence of carcinoid syndrome in a primary pediatric endobronchial carcinoid tumor. Here, we present a 14-year-old girl with chronic cough and fatigue who was found to have a primary endobronchial carcinoid tumor and concurrent carcinoid syndrome.

## 2. Case Report

A 14-year-old girl was referred to a large, regional tertiary children's hospital for evaluation of chronic cough and fatigue. She complained of a 6–8-month history of fatigue and a 2-3-month history of chronic cough that was precipitated by a viral illness. She described her cough as dry and nonproductive. A trial of inhaled corticosteroids and albuterol led to minimal improvement in her cough, and her symptoms persisted despite a five-day course of azithromycin. She also complained of low-grade fevers that occurred 1-2 times per week, night sweats, headaches, palpitations, chest pain, dyspnea, intermittent facial flushing, and diarrhea. Her past medical history was notable for mild allergic rhinitis. Her family history was significant for maternal thyroid disease but negative for other known autoimmune, oncologic, or chronic lung diseases. She lived in the Pacific Northwest, with frequent travel to the Midwest, and had exposure to horses and cows. There were no other travel or other known exposures to smoke or infectious agents. On physical examination, she had a dry cough but was not tachypneic and her oxygen saturation was 96% in room air. Additional pertinent negative exam findings included the absence of wheezes, crackles, or a prolonged expiratory phase on respiratory exam, no digital clubbing, and no cervical or supraclavicular adenopathy.

**Figure 1:**
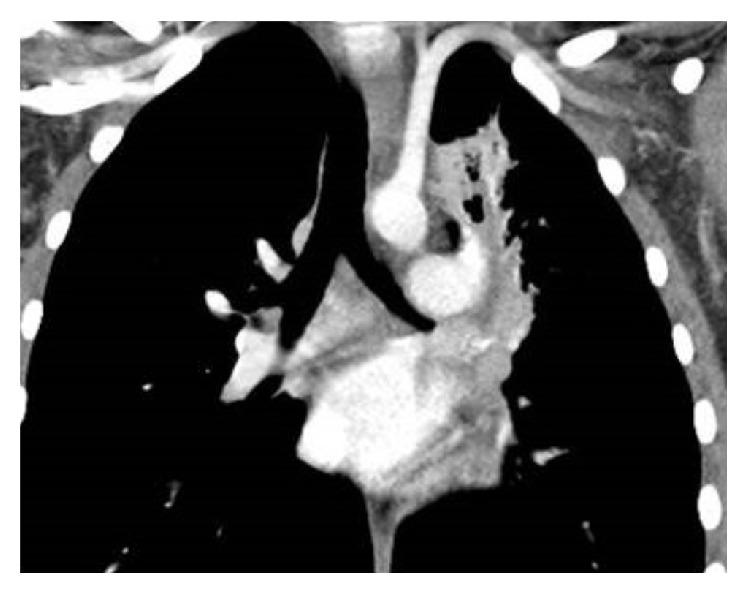
Coronal image illustrating a soft-tissue density obstructing a fluid-filled left upper lobe bronchus.

Pulmonary function testing was performed and showed no evidence of obstructive or restrictive lung disease. Additional relevant laboratory results included an elevated white blood cell count of 14.5 k/mm^3^ (normal: 4.5–11 k/mm^3^) with a neutrophil predominance, a c-reactive protein of 0.9 mg/dL (normal: 0–0.8 mg/dL), and negative virologic and serologic testing for* Epstein-Barr virus, Cytomegalovirus, Mycobacterium tuberculosis, Cryptococcus neoformans*, histoplasmosis, and* Coccidiomycosis*. A chest X-ray was obtained and was concerning for a mediastinal mass along with left upper lobe consolidation concerning for infection. A computed tomography (CT) scan of the chest demonstrated a soft-tissue density protruding into the distal aspect of the left main bronchus with fluid filling the distal left upper lobe airway (Figure 1).

## 3. Hospital Course

**Figure 2:**
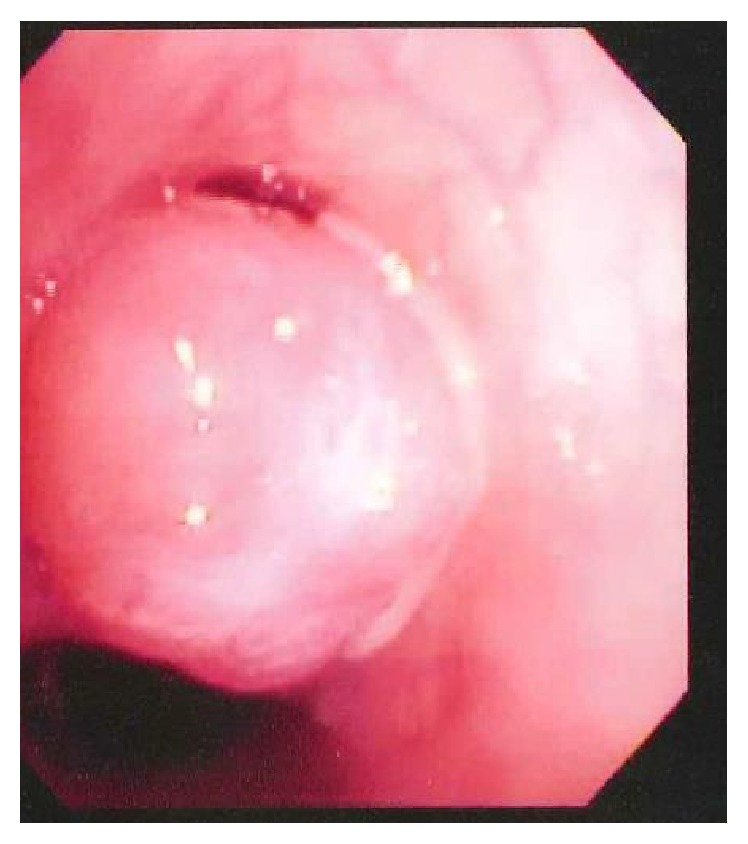
Reddish-orange smooth tissue mass obstructing the take-off of the left upper lobe bronchus.

The patient was admitted to the hospital and subsequently underwent a diagnostic flexible bronchoscopy and bronchoalveolar lavage to evaluate the lesion. The presence of a reddish-orange smooth tissue mass was noted to be completely obstructing the take-off of the left upper lobe bronchus, with purulent secretions noted around the mass (Figure 2). A bronchoalveolar lavage cultured* Haemophilus influenzae*, and she was started on antibiotics. Due to high clinical suspicion for an endobronchial carcinoid, tumor specific laboratory testing was acquired and notable for a normal neuron-specific enolase and chromogranin-A but an elevated blood serotonin level of 240 ng/mL (normal < 230 ng/mL). Pediatric thoracic surgery performed an open lobectomy and sleeve resection of the left upper lobe due to the mass location at the distal portion of the left upper lobe bronchus; a concurrent sampling lymphadenectomy was also performed. The tissue was sent to pathology which confirmed the presence of a unifocal, well-differentiated, typical endobronchial carcinoid tumor without lymph-node metastasis, extensive chronic obstructive pneumonitis, and acute bronchopneumonia. Her postoperative course was uncomplicated, and one month following the procedure she had no further cough, dyspnea, facial flushing, or fatigue.

## 4. Discussion

Endobronchial carcinoid tumors in adults are rare (<1% of all lung cancers) but are the most common intrabronchial tumor in children, accounting for 80–85% of primary malignant lung tumors [2, 4]. The most common sites for carcinoid tumors include the gastrointestinal tract and tracheobronchial tree [5]. In contrast to the adult population, pediatric patients are uniformly symptomatic, with persistent cough, wheezing, hemoptysis, and recurrent fever the most common symptoms present at time of diagnosis [2, 6, 7]. Due to these nonspecific signs and symptoms and the rarity of the condition, endobronchial tumors are frequently misdiagnosed as asthma and are subsequently found only after trials of inhaled corticosteroids or beta agonists do not improve persistent cough or wheeze. A common finding on chest radiograph is recurrent, unilateral lobar consolidation consistent with proximal occlusion of the airway. In addition, expiratory air trapping or overinflation can be produced by a ball-valve obstruction; in severe cases with complete obstruction, peripheral atelectasis and postobstructive pneumonia can be present. When intraluminal airway involvement is suspected, additional diagnostic testing with chest CT and/or direct visualization with flexible bronchoscopy is warranted.

Chest CT scans (particularly with contrast) are more useful than chest radiographs to define the tumor location and surrounding anatomy and to guide the surgical team for subsequent biopsy and/or removal. Bronchial carcinoids in children tend to arise most frequently in regions of bronchial bifurcation in the main, lobar, or segmental bronchi [8]. In order to directly visualize the lesion, flexible bronchoscopy was recommended.

When directly visualized, bronchial carcinoids typically present as smooth, pink-reddish or yellow endobronchial masses that are highly vascular [6]. Though older literature recommends against endobronchial resection or biopsy due to concerns for airway bleeding [2], more recent evidence notes that endobronchial biopsy performed at specialized centers facilitates early diagnosis without added morbidity or mortality [9, 10]. In our case and in consultation with otolaryngology and thoracic surgery, endobronchial biopsy was not pursued with concerns for airway bleeding.

Surgery represents the treatment of choice for all bronchial carcinoids; in children and adolescents, lung-sparing resections (including wedge resection, segmentectomy, or sleeve resection) are recommended when possible. Surgical resection should be combined with lymph-node dissection to confirm the absence of local regional metastases [11]. The prevalence of metastatic pediatric bronchial carcinoids is reported in a range of 5–27% [2, 6], with the liver as the most frequent site of metastasis. Experience with chemotherapy or somatostatin analogue therapy to treat metastatic disease has yielded mixed results, and there is not enough experience in children to recommend for or against these systemic treatment strategies [6, 12]. Overall survival for children and adolescents following resection is 90% [2, 4] with encouraging disease-free survival for 10 and 20 years of 96% and 94%, respectively [6]. Screening for recurrence is not established and without guidelines from the National Comprehensive Cancer Network.

Carcinoid syndrome—characterized by flushing, palpitations, wheezing, shortness of breath, and diarrhea [3]—is caused by serotonin release from the tumor and is present in up to 30% of carcinoids. It is extremely rare (0.7% of all cases) in conjunction with adult bronchial carcinoid tumors [6] and to our knowledge has been reported in one prior pediatric endobronchial carcinoid tumor [13]. In addition to the characteristic signs and symptoms, other objective findings that support concurrent carcinoid syndrome include elevations in chromogranin A, neuron-specific enolase, and serotonin in the blood [6] and elevation in the 24-hour urinary collection of 5-hydroxyindolacetic acid (5-HIAA) [3]. If there is clinical suspicion of metastatic spread, a radio-labelled somatostatin analogue scintigraphy scan remains the gold standard for confirming the location of any functioning neuroendocrine tumor tissue, since all neuroendocrine tumors express somatostatin receptors [3]. Finally, bronchial carcinoids can rarely cause Cushing's syndrome due to ectopic production of adrenocorticotropic hormone (ACTH), and symptoms are seen in 1-2% of these patients [14].

Our patient presented with signs and symptoms consistent with carcinoid syndrome, including facial flushing, dyspnea, chest pain, diarrhea, and palpitations. In addition, although her serum chromogranin A and neuron-specific enolase were within normal limits, her serum serotonin level was elevated. Finally, one month following surgical removal, our patient no longer had any systemic or respiratory complaints.

Carcinoid syndrome is extremely rare found in conjunction with pediatric endobronchial tumors and should be considered in the setting of persistent systemic symptoms with recurrent pneumonia. Initial management consists of referral to a pediatric pulmonologist and pediatric thoracic surgeon to expedite diagnosis and surgical resection. Early diagnosis limits risk of metastatic spread and facilitates disease-free survival.
